# Platelet‐activating factor antagonist‐based intensive antiplatelet strategy in acute ischemic stroke: A propensity score matched with network pharmacology analysis

**DOI:** 10.1111/cns.14331

**Published:** 2023-07-12

**Authors:** Xiaoyan Han, Youjia Li, Xuemin Chen, Dong Pan, Junning Mo, Jiaming Qiu, Yi Li, Yan Chen, Yan Huang, Qingyu Shen, Yamei Tang

**Affiliations:** ^1^ Department of Neurology First People's Hospital of Zhaoqing Zhaoqing People's Republic of China; ^2^ Department of Neurology, Sun Yat‐sen Memorial Hospital Sun Yat‐sen University Guangzhou People's Republic of China; ^3^ Guangdong Medical University Zhanjiang People's Republic of China; ^4^ Guangdong Provincial Key Laboratory of Malignant Tumor Epigenetics and Gene Regulation, Sun Yat‐sen Memorial Hospital Sun Yat‐sen University Guangzhou People's Republic of China; ^5^ Guangdong Province Key Laboratory of Brain Function and Disease, Zhongshan School of Medicine Sun Yat‐Sen University Guangzhou People's Republic of China

**Keywords:** inflammation, intensive antiplatelet strategy, ischemic stroke, platelet‐activating factor receptor antagonist, propensity score matching

## Abstract

**Background:**

Diterpene ginkgolides meglumine injection (DGMI) is a platelet‐activating factor receptor (PAFR) antagonist that can be used to treat acute ischemic stroke (AIS). This study evaluated the efficacy and safety of an intensive antiplatelet strategy based on PAFR antagonists and explored the underlying mechanisms of PAFR antagonists in AIS treatment.

**Methods:**

This is a retrospective study applying propensity score methods to match AIS patients treated with DGMI to nontreated patients. The primary outcome was functional independence (modified Rankin Scale [mRS] 0–2) at 90 days. The safety outcome was bleeding risk. We used McNemar test to compare the efficacy outcome. Subsequently, the network pharmacology analysis was performed.

**Results:**

161 AIS patients treated with DGMI in the study were matched with 161 untreated patients. Compared with untreated patients, DGMI‐treated patients had a significantly higher rate of mRS ranking 0–2 at 90 days (82.0% vs. 75.8%, *p* < 0.001), without increased risk of bleeding. The gene enrichment analysis showed that the overlap genes of DGMI targeted and AIS‐related enriched in thrombosis and inflammatory‐related signaling pathways.

**Conclusions:**

An intensive antiplatelet strategy of DGMI plus traditional antiplatelet agents is effective in treating AIS and may work by mediating post‐stroke inflammation and thrombosis.

## Graphical abstract

Diterpene ginkgolides meglumine injection (DGMI) plus traditional antiplatelet agents is an effective and safe intensive antiplatelet strategy in treating acute ischemic stroke. And it may work by mediating post‐stroke inflammation and thrombosis‐related signaling pathways.

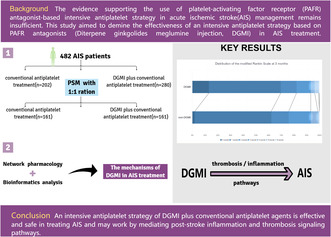

## 1 INTRODUCTION

Acute ischemic stroke (AIS) sufferers are at high risk of subsequent disability and mortality, particularly when it is caused by arterial occlusion.^1^ Intravenous thrombolysis and endovascular thrombectomy are the most recommended management for the acute phase of AIS but are time‐critical.^2^ However, a proportion of AIS patients still did not achieve complete recanalization,^3^ and approximately 40%–50% of successful reperfusion patients retained unfavorable outcomes.^4^, ^5^ The differences in treatment outcomes in patients with AIS may be attributed in part to the ongoing thromboembolic formation and early arterial re‐occlusion by platelet activation and subsequent platelet aggregation, irrespective of the patient receiving revascularization therapy.^6^, ^7^ Therefore, antiplatelet agents remain the cornerstone among the early treatments of AIS. Studies from the last decade have shown that an intensive antiplatelet strategy improves outcomes for certain stroke subtypes.^8^, ^9^, ^10^, ^11^, ^12^ Given the balance between the benefits of intensive antiplatelet therapy and an increased risk of bleeding, the previous guidelines only recommend the short‐term use of combined antiplatelet therapy with aspirin and clopidogrel following a minor ischemic stroke or a high‐risk transient ischemic attack (TIA).^13^ Therefore, exploring safe and effective antithrombotic therapies against early platelet aggregation in AIS has received great concern.

Platelet‐activating factor (PAF) acts as a potent phospholipid mediator of platelet aggregation and proinflammatory signaling, which activated platelets independent of the cyclooxygenase pathway.^14^, ^15^, ^16^ Diterpene ginkgolides meglumine injection (DGMI), a novel PAF antagonist, is an extract from Ginkgo leaves, comprising mainly of ginkgolide A, B, and K (≥98%). It has been previously reported to be the highly selective antagonists of the PAF receptor (PAFR).^17^ In terms of the mechanism of drug‐targeted action, DGMI may be a promising antiplatelet therapy for AIS independent of Aspirin and P2Y12 receptor inhibitors.

A multicenter, randomized controlled clinical trial (RCT) RCT has shown ginkgolide plus aspirin to be more effective than aspirin monotherapy for AIS patients with intracranial artery stenosis (ICAS).^18^ However, an intensive antiplatelet strategy combining PAF antagonists with guideline‐recommended antiplatelet regimens has not been validated in real‐world AIS study. Therefore, to investigate the efficacy and safety of an intensive antiplatelet regimen based on PAFR antagonists in AIS, we compared the different antiplatelet routing: DGMI plus conventional antiplatelet strategy versus conventional antiplatelet strategy for AIS patients without vessel reperfusion treatment. Furthermore, the underlying antithrombosis and anti‐inflammatory mechanisms of DGMI as a PAFR antagonist in AIS treatment were also explored.

## 2 METHOD

### 2.1 Study populations selection

This was a retrospective study in compliance with STROBE criteria,^19^ which involved consecutive patients with AIS hospitalized in the Zhaoqing First People's Hospital from June 2017 to May 2022. The inclusion criteria were as follows: (a) over the age of 18; (b) admitted to hospital within 48 h of onset of symptoms; (c) diagnosed with AIS based on clinical symptoms, brain magnetic resonance imaging (MRI) manifestations, as well as the criteria of the Trial of Org 10172 in Acute Stroke Treatment (TOAST)^20^; (d) treated with the standard antiplatelet protocol (Aspirin/Clopidogrel monotherapy, or Aspirin plus Clopidogrel) recommended by the Chinese guideline^21^; (e) having received platelet function testing after 7 days of antiplatelet treatment with a documented 3‐month follow‐up of onset. Patients were excluded in the presence of any of the following conditions: (a) had incomplete medical records affecting subsequent analysis; (b) had other medications within hospitalization affecting hematological coagulation, such as argatroban, tirofiban, cilostazol, heparin, warfarin, dabigatran, and factor Xa inhibitors; (c) had a history of malignancies, systemic inflammatory disorders, hematological disorders, severe liver, kidney, or heart dysfunction; (d) had been undergoing intravenous thrombolysis or endovascular thrombectomy after the admission. This study was approved by the Zhaoqing First People's Hospital Ethics Committee (No. of approval: B2021‐11‐02). The patient's written informed consent was waived because all data analyzed in this study were anonymized.

### 2.2 Clinical data

Baseline demographics, medical history (diabetes mellitus, hypertension, hyperlipidemia, ischemic stroke, and hyperlipidemia), smoking status, National Institute of Health stroke scale (NIHSS) score at admission, modified Rankin Scale (mRS) prior to onset, and stroke subtypes were included. The routine blood examinations were conducted on admission within the first 24 h. Stroke etiology was categorized following TOAST classification.^20^ NIHSS score was employed to assess the severity of stroke.^22^ Patient comorbidities, stroke severity, treatment information, safety, and efficacy outcomes were recorded as well.

### 2.3 Antiplatelet intervention

The administration of DGMI was at the discretion of the attending neurologist. According to the antiplatelet regimen administered in the 7 days after admission, the participants were assigned to two groups: DGMI plus conventional antiplatelet‐treated group and conventional antiplatelet‐treated group. In general, the DGMI treatment was treated immediately after admission as follows: 25 mg of DGMI (Jiangsu Kanion Pharmaceutical Co., Ltd.) diluted in 250 mL of 0.9% NaCl injection was intravenously injected daily for 7 days. The conventional antiplatelet regiment was defined as the aspirin (100 mg oral daily)/clopidogrel (75 mg oral daily) monotherapy or dual antiplatelet therapy (DAPT) recommended by current guidelines on an individual basis. In addition, all patients enrolled were treated individually according to the guidelines,^21^ such as antihypertension, lipid and glucose‐lowering, and neuroprotection for AIS.

### 2.4 Efficacy and safety outcomes

The primary outcome was functional independence (90 days mRS 0–2). The secondary outcomes included freedom from disability (90‐days mRS 0–1), and the incidence of early neurological deterioration (END). END was measured as an increase of 4 or more points in NIHSS score within 7 days after admission compared with baseline.^23^ The primary safety outcome was symptomatic intracranial hemorrhage (sICH), defined as computed tomography (CT) evidence of intracranial hemorrhage associated with a 4‐point increase in NIHSS score.^24^ Other safety outcomes included the presence of asymptomatic intracranial hemorrhage (aICH) without clinical worsening despite evidence of hemorrhagic transformation on the brain CT,^25^ and the presence of any bleeding events. CT imaging obtained after treatment was in response to adverse patient clinical changes per the discretion of the treating team or patients who had an NIHSS ≥4‐point worsening of NIHSS at 24 h. Clinical assessment evaluation at 90 days follow‐up was performed by a neurologist or a trained specialist nurse who is certified in mRS assessment from the senior stroke center through face‐to‐face or telephone interviews.

### 2.5 Assessment of platelet function

This study included platelet function testing results to assess the PAFR antagonist synergistic antiplatelet effect in AIS patients. Platelet reactivity was assessed by the thromboelastography (TEG) platelet mapping (BVCA‐VIII, Bring Biology) at 7 ± 2 days after the initiation of antiplatelet therapy. Seven TEG parameters were examined: (1) reaction time (R, min), signifying the time from clotting factor activation to initial clot formation; (2) coagulation time (K, min), signifying the time from clot formation to reach a 20 mm amplitude; (3) angle (α, degree), signifying the rate of clot formation; (4) maximum amplitude (MA), signifying the maximum strength of the clot; (5) MAADP, signifying the strength of the clot induced by adenosine diphosphate (ADP); (6) arachidonic acid (AA) inhibition rate (AA%), signifying the reaction to aspirin to aspirin; and (7) ADP inhibition rate (ADP%), signifying the reaction to clopidogrel.

### 2.6 Identification of the potential mechanism of DGMI in AIS through bioinformatics and network pharmacology

To find the differentially expressed genes (DEGs) of AIS, we downloaded the gene expression profile from the Gene Expression Omnibus (GEO) (https://www.ncbi.nlm.nih.gov/geo/): 63 blood samples (39 samples with AIS and 24 health controls) in GSE16561.^26^ We used the “limma” R package to normalize the expression data (Figure S1) and to identify the DEGs between AIS and the control blood samples with a threshold of |logFC| >0.5 and an adjusted *p* value <0.05

We input DGMI active compounds (Ginkgolides A, B, and K) into the PubChem Database **(** https://pubchem.ncbi.nlm.nih.gov) to achieve their smile numbers.^27^ The smiles numbers were imported into SwissTargetPrediction (http://www.swisstargetprediction.ch) to predict the drug‐target,^28^ and then they were imported into UniProt (http://www.uniprot.org/) to obtain the official gene symbol name, setting the organism “Homo sapiens”.^29^ Using these web tools, we acquired predicted drug‐target genes of each compound. After merging the duplicate data, we chose the target genes as potential targets of DGMI. Additionally, we calculated and made Venn diagrams for overlap genes of AIS‐DEGs and DGMI‐target genes using the Sangerbox 3.0 tool (http://www. http://sangerbox.com/home.html).^30^

To further indicate the biological processes (BPs) and signaling pathways that involve DGMI in the treatment of AIS, the gene ontology (GO) and Reactome pathways enrichment analysis were performed by submitting overlapping genes of DGMI and AIS‐DEGs, based on the R “clusterProfiler” package. The cut‐off criteria for enrichment analysis were *p* < 0.05 and false discovery rate (FDR) < 0.05.

### 2.7 Statistical analysis

Results are presented as mean ± standard deviation (SD) for normally distributed continuous variables, as median (interquartile range [IQR]) for non‐normally distributed data, and as number (%) for categorical variables. The Shapiro–Wilk normality test was performed on all continuous variables before analysis. The missing data were imputed using the k‐Nearest Neighbor (kNN) algorithm. Patients were stratified into two groups depending on treatment with DGMI or not. Given the selection bias inherent to retrospective studies and the confusion bias, we used propensity score matching (PSM) applying a logistic regression model for balancing the distribution of the baseline characteristics and potential confounders between groups, including demographic data (age, sex), previous history (history of diabetes mellitus, hypertension, hyperlipidemia, ischemic stroke, and smoking status), baseline clinical characteristic (TOAST subtypes, carotid atherosclerosis, and blood white blood cell [WBC], serum creatinine [Scr], blood cholesterol [CHOL], glycated hemoglobin [HbA1c], prothrombin time [PT], fibrinogen [Fib], NIHSS at admission, mRS before admission, and antiplatelet treatment strategy). Using the R “MatchIt” package, patients were matched 1:1 using the nearest neighbor matching with a caliper of 0.05 on a logit scale. Figure S2 displays the distribution of the propensity score before and after matching in DGMI‐treated group and un‐treated group. There is adequate overlap between the two treatment groups. Characteristics of patients included in generating the propensity scores were compared before and after matching using a standardized differences.^31^ After matching, binary outcomes were compared using McNemar test, and TEG parameters were compared using paired t tests. Analyses were performed by R software (version 4.1.5, R Foundation), and a *p* < 0.05 was considered as statistical significance.

The data used and analyzed during the current study are available from the corresponding author on reasonable request.

## 3 RESULTS

**FIGURE 1:**
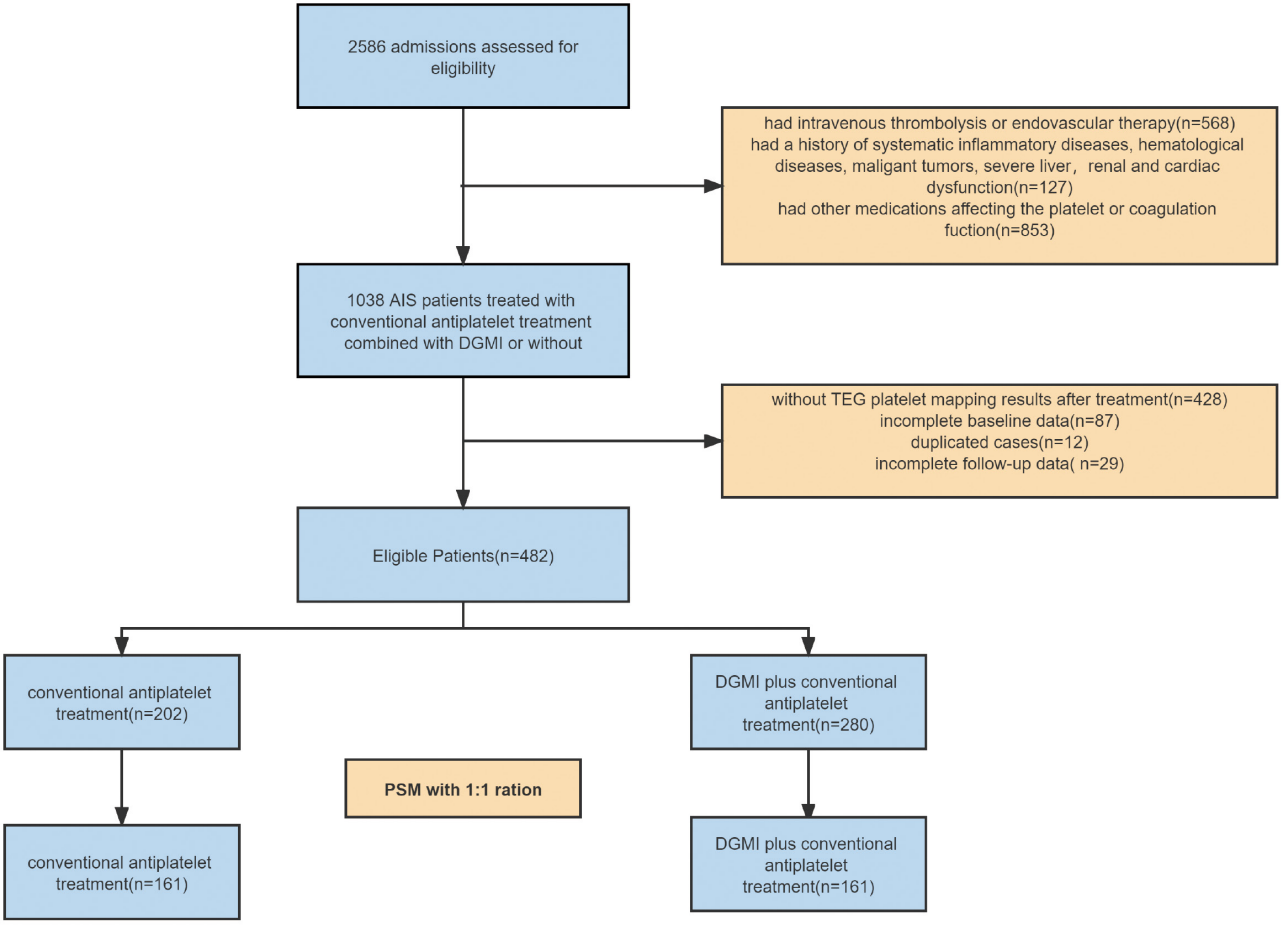
Flow chart of admission record selection. AIS, acute ischemic stroke; DGMI, diterpene ginkgolides meglumine injection; PSM, propensity score matching; TEG, thromboelastography.

In total, 2586 AIS hospitalization records were screened for eligibility, and 482 admissions met the selection criteria and were eligible for the analysis (Figure 1). Patients prescribed DGMI (*n* = 280) were slightly younger, had a higher prevalence of smoking, stroke etiology of large‐artery atherosclerosis (LAA), had a higher PT level and a lower Scr level at baseline, and were more likely to receive DAPT during hospitalization before PSM (Table 1).

**TABLE 1:** Baseline patient characteristics in patients treated with and without DGMI before and after Propensity Score Matching.

Characteristic		Full cohort			Propensity score matched cohort		
		Non‐DGMI (*n* = 202)	DGMI (*n* = 280)	Std diff^a^	Non‐DGMI (*n* = 161)	DGMI (*n* = 161)	Std diff^a^
Demography							
Age, years, mean (SD)	66.36 (10.86)	67.39 (10.34)	65.61 (11.17)	0.159	66.5 (10.4)	66.4 (11.3)	0.011
Sex, Male, *n* (%)	335 (69.5)	137 (67.8)	198 (70.7)	0.064	109 (67.7)	108 (67.1)	0.014
Vascular risk factors, *n* (%)							
Current smoking	210 (43.6)	79 (39.1)	131 (46.8)	0.154	67 (41.6)	76 (47.2)	0.112
Previous diabetes	99 (20.5)	44 (21.8)	55 (19.6)	0.054	38 (23.6)	33 (20.5)	0.078
Previous hypertension	283 (58.7)	120 (59.4)	163 (58.2)	0.024	96 (59.6)	98 (60.9)	0.025
Previous hyperlipidemia	20 (4.1)	8 (4.0)	12 (4.3)	0.016	7 (4.3)	6 (3.7)	0.031
Previous ischemic stroke	111 (23.0)	47 (23.3)	64 (22.9)	0.010	41 (25.5)	34 (21.1)	0.104
Baseline characteristics							
WBC, Median (IQR), ×10^9^/L	8.5 (6.8, 10.6)	8.5 (6.8, 10.6)	8.5 (6.8, 10.6)	0.156	8.2 (6.7, 10)	7.8 (6.6, 10.1)	0.049
PT, Median (IQR), g/L	12.1 (11.4, 12.9)	12.4 (11.5, 13.1)	11.8 (11.3, 12.6)	0.417	12.1 (11.4, 12.9)	12.1 (11.4, 13.1)	0.114
Fib, Median (IQR), g/L	3.22 (2.79, 3.90)	3.28 (2.87, 3.98)	3.17 (2.73, 3.78)	0.195	3.2 (2.9, 3.9)	3.2 (2.8, 3.8)	0.041
Scr, median (IQR), μmol/L	70.2 (43, 88.6)	72.4 (53.2, 92.7)	66.2 (39.7, 84.8)	0.253	71.6 (46.8, 87.9)	66.7 (40.3, 83)	0.127
HbA_1c_, median (IQR), mmol/L	6 (5.6, 6.9)	6 (5.6, 7.1)	6.1 (5.6, 6.8)	0.039	6 (5.5, 7.4)	6 (5.6, 7)	0.108
CHOL, median (IQR), mmol/L	4.8 (4.1, 5.5)	4.6 (4, 5.6)	4.8 (4.2, 5.5)	0.061	4.7 (4, 5.6)	4.7 (4.2, 5.4)	0.054
NIHSS at admission, median (IQR)	4.00 (3.0, 7.00)	4.0 (3.0, 7.0)	4.0 (3.0, 6.0)	0.079	4 (3, 7)	4 (3, 6)	0.055
MRs before admission, median (IQR)	0.0 [0.0, 0.0]	0.0 (0.0, 0.00)	0.0 (0.0, 0.00)	0.163	0.0 (0.0, 0.00)	0.0 (0.0, 0.00)	0.029
Stroke etiology (TOAST), *n* (%)							
LAA	316 (65.6)	126 (62.4)	190 (67.9)	0.117	111 (68.9)	104 (64.6)	0.093
CE	82 (17.0)	33 (16.3)	49 (17.5)	0.031	26 (16.1)	26 (16.1)	<0.001
SAA	64 (13.3)	33 (16.3)	31 (11.1)	0.168	19 (11.8)	26 (16.1)	0.139
Others	20 (4.1)	10 (5.0)	10 (3.6)	0.074	5 (3.1)	5 (3.1)	<0.001
Carotid atherosclerosis, *n* (%)							
Non	22 (4.6)	7 (3.5)	15 (5.4)	0.084	6 (3.7)	10 (6.2)	0.110
Carotid plaque	309 (64.1)	132 (65.3)	177 (63.2)	0.044	104 (64.6)	99 (61.5)	0.064
Carotid severe stenosis	151 (31.3)	63 (31.2)	88 (31.4)	0.005	51 (31.7)	52 (32.3)	0.013
Antiplatelet treatment, *n* (%)							
Aspirin	200 (41.5)	94 (46.5)	106 (37.9)	0.179	68 (42.2)	64 (39.8)	0.051
Clopidogrel	94 (19.5)	32 (15.8)	62 (22.1)	0.152	30 (18.6)	31 (19.3)	0.015
Aspirin plus clopidogrel	188 (39.0)	76 (37.6)	112 (40.0)	0.049	63 (39.1)	66 (41)	0.051

Abbreviations: CE, cardioembolism; CHOL, total cholesterol; DGMI, Diterpene ginkgolides meglumine injection; Fib, Fibrinogen; HbA_1c_, glycated hemoglobin; IQR, interquartile ranges; large‐artery atherosclerosis; MRs, modified Rankin score; NIHSS, National Institute of Health Stroke Scale; Others: include SOE, stroke of other determined etiology and SUE, stroke of undetermined etiology; PT, prothrombin time; SAA, small‐vessel occlusion; Scr, serum creatinine; SD, standard deviation; Std Diff, standardized difference. Carotid severe stenosis was defined as the 70%–99% stenosis or occlusion of at least one of the internal carotid arteries in the present study; TOAST, the trial of ORG 10172 in Acute Stroke Treatment; WBC, white blood cell.

^a^ Standardized difference of <0.1 indicates adequate matching.

### 3.1 Clinical outcomes

**FIGURE 2:**
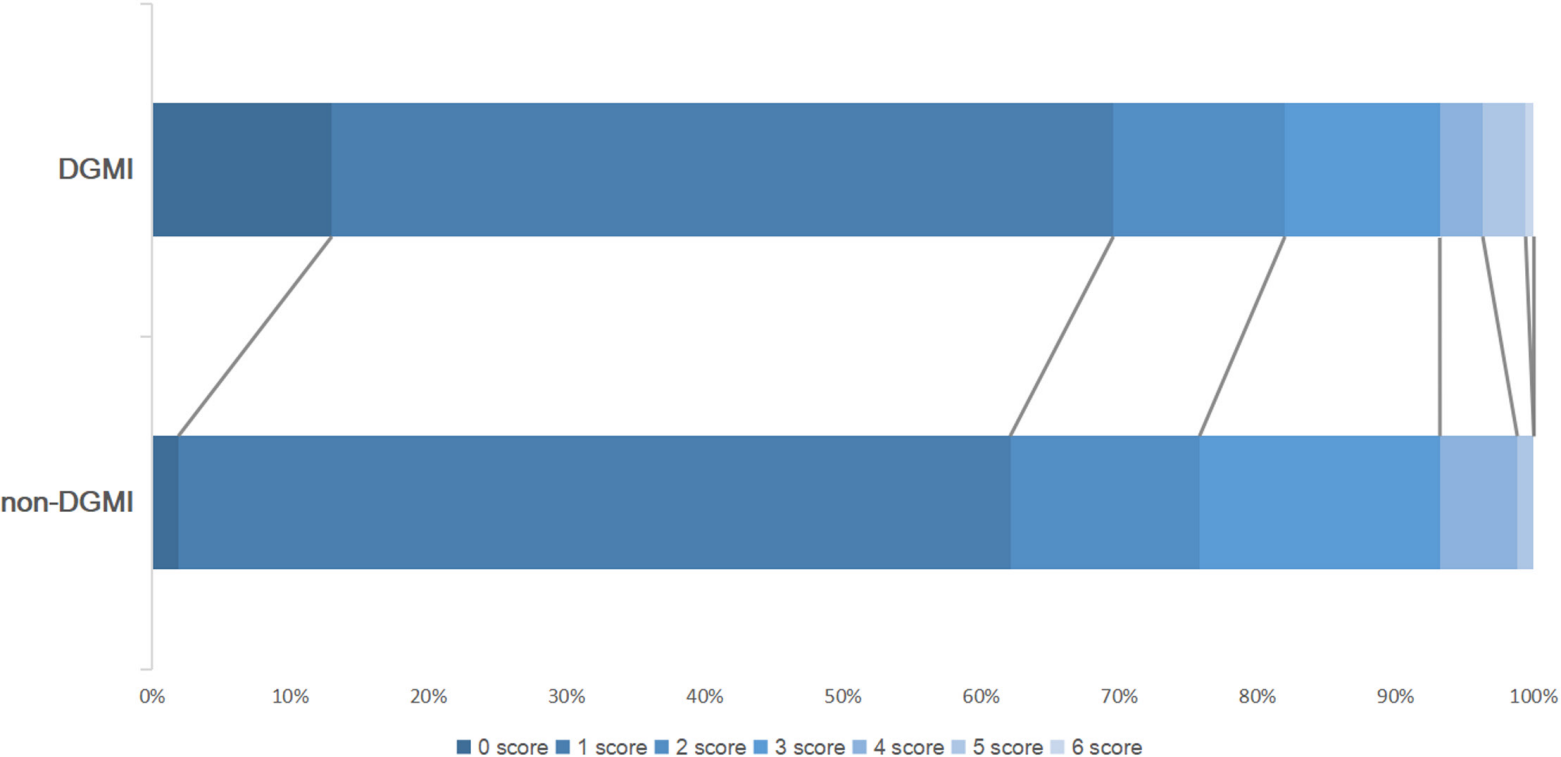
Distribution of the modified Rankin Scale at 3 months between the two groups. DGMI, diterpene ginkgolides meglumine injection.

After 1:1 propensity score matching, a total 161 of patients were allocated in each group and the majority of baseline characteristics of confounders were well balanced with a standardized difference <0.1 (Table 1). The main cause of END in our study was stroke progression, symptom fluctuations, and brain edema. When compared with the conventional antiplatelet group, the DGMI plus conventional antiplatelet group had a significantly higher proportion of mRS ranking 0–2 at 90 days (82.0% vs. 75.8%, *p* < 0.001), a significantly higher proportion of mRS ranking 0–1 at 90 days (69.6% vs. 62.1%, *p* < 0.001), and a lower incidence of END (10.6% vs. 12.4%, *p* < 0.001; Table 2, Figure 2). There was no significant difference in the proportion of aICH events (2/161 [1.2%] vs. 3/161 [1.8%]), or the proportion of other bleeding events (1.8% vs. 1.8%) between the two groups. Besides, the sICH event was not observed in the two groups.

**TABLE 2:** Outcome for patients with ischemic stroke treated with DGMI or without.

Outcomes	Non‐DGMI (*n* = 161)	DGMI (*n* = 161)	*p*‐value
Efficacy outcomes, *n* (%)			
3‐month mRS 0–2	122 (75.8)	132 (82)	<0.001
3‐month mRS 0–1	100 (62.1)	112 (69.6)	<0.001
Early neurology deterioration	20 (12.4)	17 (10.6)	<0.001

Abbreviations: DGMI, diterpene ginkgolides meglumine injection; mRS, modified Rankin score.

No significant difference was observed between the two groups in levels of all TEG variables reflecting coagulation and platelet function after antiplatelet therapy (all *p* > 0.05, Table S1).

### 3.2 Bioinformatics and network pharmacology analysis

As a result, a total of 669 AIS‐related DEGs were screened out from GSE16561 dataset. The result included 504 down‐regulated and 165 upregulated genes and is shown in Figure S3A. A total of 152 target genes of each candidate DGMI compound were obtained and we defined 14 overlapping genes between the targets of DGMI and AIS‐related DEGs (Figure S3B). To indicate the specific circumstances of the association of each active constituent of DGMI with the potential targets, a “Drug‐Active Constituent‐Potential Target” interactive network is displayed in Figure S3C.

**FIGURE 3:**
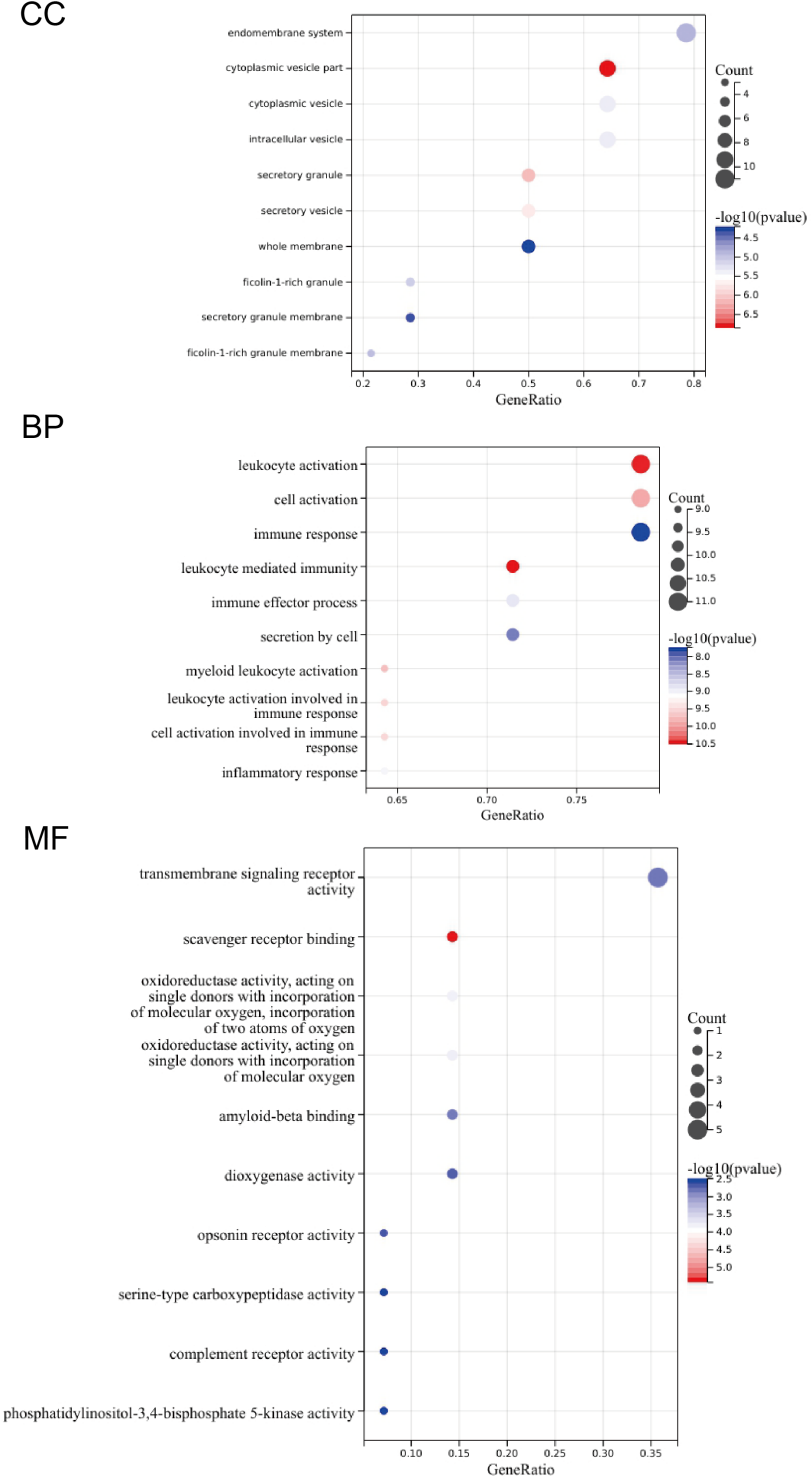
Biological functions based on gene ontology (GO) analysis of DGMI‐AIS‐related hub genes. Advanced bubble chart shows the Top 10 significance in GO enrichment items of hub genes in three functional groups: biological process (BP), cell composition (CC), and molecular function (MF). The *x*‐axis label represents the gene ratio (Rich Factor) and the *y*‐axis label represents GO terms. AIS, acute ischemic stroke; DGMI, diterpene ginkgolides meglumine injection.

The overlapping genes represent targets for the combined application of DGMI for the treatment of AIS and were analyzed to obtain the gene functional enrichment analysis. The top 10 terms for biological process BP, cell composition (CC), molecular function (MF) enrichment of GO were listed in Figure 3. The overlapping genes were most significantly enriched in complement receptor‐mediated signaling pathway, regulation of inflammatory response for BP, ficolin‐1‐rich granule for CC, complement receptor activity, oxidoreductase activity for MF. The top 20 terms enriched in GO enrichment analysis are listed in Table S2.

**FIGURE 4:**
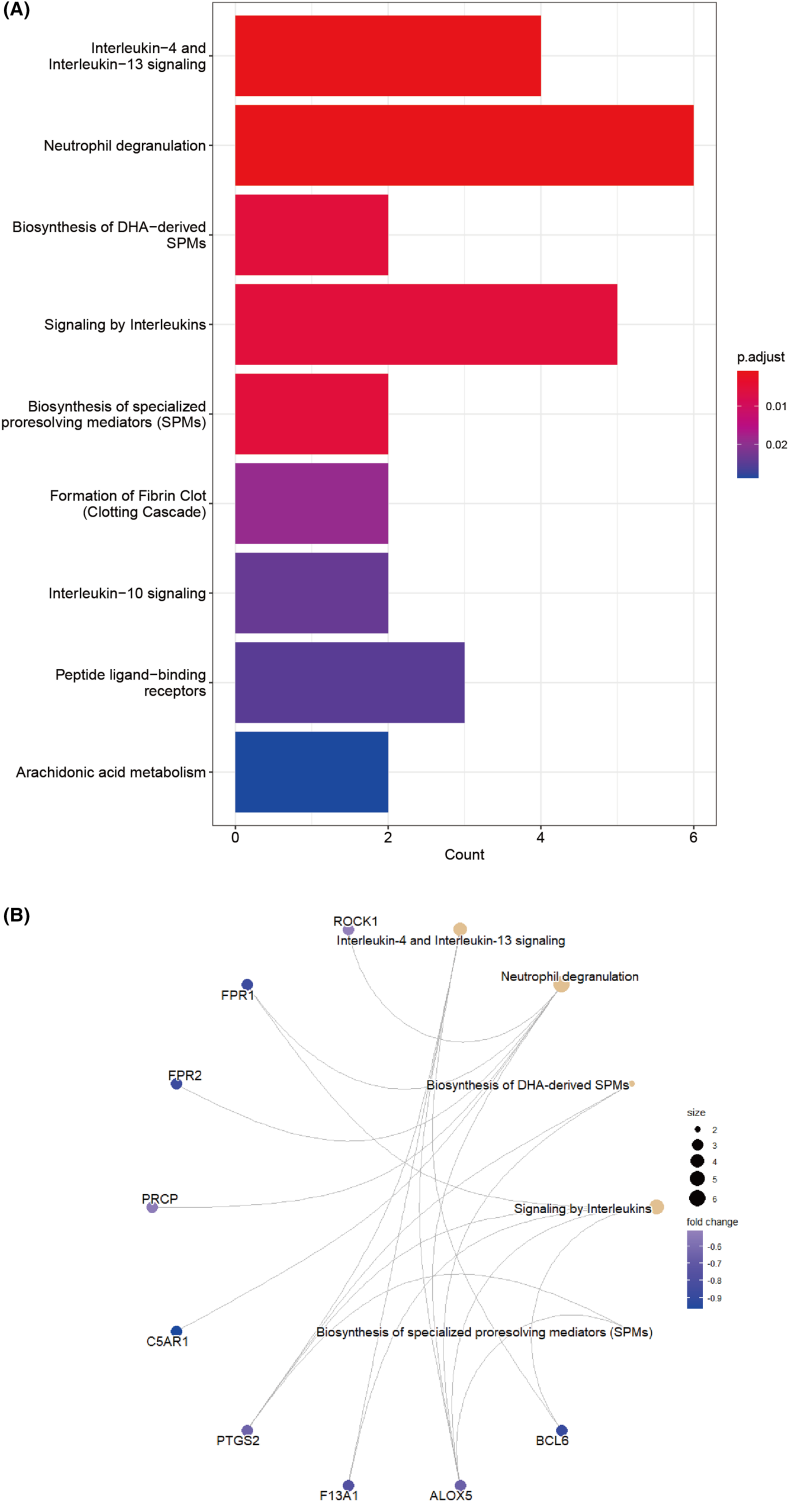
Reactome pathways enrichment analysis and functional association networks between signature genes and pathway signaling. (A) Top 10 Reactome pathways with high count, (B) functional association networks between AIS‐DGMI overlap genes and enriched pathways. AIS, acute ischemic stroke; DGMI, diterpene ginkgolides meglumine injection.

Reactome pathways enrichment analysis showed that the overlapping genes were primarily enriched in interleukin‐4 and interleukin‐13 signaling, neutrophil degranulation, biosynthesis of DHA‐derived SPMs, signaling by interleukins (Figure 4, Table S3).

## 4 DISCUSSION

Our study has demonstrated the effectiveness of DGMI plus conventional antiplatelet regimens over the current conventional antiplatelet regimens for AIS. As far as we know, this is the first study to investigate the role of the PAFR antagonist‐based intensive antiplatelet strategy in AIS treatment. Using PSM analysis, we found that short‐term application of DGMI plus conventional antiplatelet regimen was associated with a better 90‐day prognosis and fewer END risks without an increased risk of bleeding. Furthermore, we use the bioinformatics and network pharmacology analyses to provide insights into the potential mechanisms by which the DGMI exerts the effects on thrombosis and inflammation pathways in AIS.

As new promising antithrombotic agents, the therapeutic effect of PAFR antagonists on AIS has been recently explored. The Ginkgolide in Ischemic Stroke patients with large‐Artery Atherosclerosis (GISAA) trial has shown that DAPT (Ginkgolide plus aspirin) is beneficial in reducing recurrent stroke in AIS with intracranial artery stenosis patients.^18^ The post hoc analyses of GISAA also confirmed the safety and efficacy of the Ginkgolide‐based DAPT strategy in elderly patients.^16^ The results of the POINT and CHANCE trials have demonstrated the superiority of short‐term DAPT to aspirin alone in minor ischemic stroke and TIA,^8^, ^10^ but it is unclear whether an intensive antiplatelet strategy (DAPT or triple antiplatelet therapy) is beneficial to patients with other stroke subtypes. One of the major limitations of intensive therapy in AIS is that the increased risk of bleeding events might offset the efficacy of the antithrombotic treatments. Similar to previous studies, our study has established the safety of PAFR antagonists combined with other antiplatelet drugs in AIS, even in patients with more severe stroke or with DAPT.

Although previous studies suggested that PAFR antagonists could inhibit platelet aggregation in vivo and in vitro,^32^, ^33^, ^34^ data available investigating the effects of PAFR antagonists on coagulation and platelet function in patients with AIS are scarce. The GISAA study found that the effect of Ginkgolide in AIS was associated with the PAF pathway but not with the ADP or thromboxane A2 (TxA2) pathways.^18^ Similarly, the current study found that neither AA‐mediated nor ADP‐mediated platelet reactivity tested by TEG differed significantly between the group treated with DGMI and that with not. It suggested that the therapeutic benefit of DGMI might not come from improving antiplatelet drug resistance to aspirin or clopidogrel. The underlying synergistic antithrombotic mechanisms of DGMI and conventional antiplatelet regimens remain unknown. The Reactome pathways enrichment analysis of AIS‐DEGs and DGMI overlaps genes significantly enriched in the formation of fibrin clots and arachidonic acid metabolism signaling pathways, implying that these thrombosis‐related signaling pathways could be the mechanisms of the DGMI‐mediated AIS antithrombotic process. Based on the results of the current study, we speculated that DGMI‐based intensive antiplatelet regimens do not increase bleeding risks since DGMI selectively impedes thrombus formation in the brain in AIS without affecting systematic coagulation or platelet function.

Experimental evidence suggested that PAFR antagonists may exert neuroprotective effects in ischemic stroke mainly through anti‐apoptosis,^35^ anti‐inflammation,^36^ turnover of harmful autophagy,^37^ pro‐angiogenic activity,^38^ and attenuating neuronal pyroptosis.^39^ Our work integrating bioinformatics combined with network pharmacology approaches further offers new mechanistic insight into our observations. According to the functional enrichment results, DGMI's therapeutic effect was directed through mediating inflammatory‐related signaling pathways, such as IL‐4 and IL‐13 signaling. Microglia/macrophages are highly plastic immune cells with different polarizations and can be either pro‐inflammatory (M1 polarization) or anti‐inflammatory (M2 polarization) when responding to specific microenvironmental signals after cerebral ischemia.^40^ IL‐4 production in the central nervous system is essential for the induction of M2 polarization, with the subsequent expression of different molecules related to scavenger receptors and pro‐angiogenic factors, such as mannose receptors, ectin‐1, and arginase, which perform neuroprotective effect.^41^, ^42^ IL‐13 acts as an anti‐inflammatory cytokine and plays a role in regulating the polarization of microglia/macrophages.^43^ At early cerebral ischemia, microglia, and macrophages can activate T cells and produce IL‐17.^44^ Previous studies have found that IL‐17 exerts a pro‐inflammatory cytokine effect by activating NF‐κB/MAPK pathway signaling and a decrease in serum IL‐17A in patients with good clinical outcomes after DGMI treatment.^32^ In addition, XQ‐1H, a new derivative of ginkgolide B, was found to protect mice from ischemic injury by regulating microglia polarization via the PPARγ pathway.^45^ These findings suggested that DGMI reduced inflammation in AIS patients by modulating the balance of pro‐ and anti‐inflammatory microglia polarization.

Growing evidence suggests that inflammation is strongly associated with thrombosis through VWF‐mediated reactivity, which interacts with tissue factor‐initiated fibrin formation and may lead to extensive thrombotic disease.^46^ Functional and genetic evidence of DGMI against AIS indicated enrichment in multiple pathways mainly implicated in thrombosis and inflammation. Similar to VWF, PAF might be the link between inflammation and thrombosis, which makes it an ideal target for AIS therapy.

The strengths of the study include the observational study design using the PSM method and in‐depth mechanism of investigation that utilizes transcriptomics with network pharmacology. However, several limitations are noteworthy. First of all, this study is a retrospective observational study that enrolled participants from a Chinese monocenter, which could result in selection bias. Second, because the antiplatelet regimen administration route was based on physicians’ decision, the potential for confounding associated with clinical outcomes might not be balanced. However, the inclusion of variables such as NIHSS at admission and stroke etiology could improve this limitation, and statistical models can take the effect of latent variables into account in statistical analyses. Furthermore, although we evaluated platelet function after DGMI treatment, we did not have the dynamic measurement of PAF, the influence of DGMI plus conventional antiplatelet agents on PAF‐dependent platelet aggregation after AIS was not available in the present study. In addition, Figure 2 showed that when compared with the conventional antiplatelet group, the DGMI plus conventional antiplatelet group had a higher proportion of mRS ranking 5–6 at 90 days (6 [3.7%] vs. 2 [1.2%]). Even though hemorrhagic transformation was not the reason for a higher proportion of mRS 5–6 in the DGMI group, the mechanism of DGMI‐based antiplatelet therapy could bring significant beneficial effects (improving functional outcomes at 3 months) to AIS patients, but without reducing the rate of mRS 5‐6 at 3 months when compared with non‐DGMI group remains unknown. Further studies focus on improving the above deficiencies and the interplay mechanism of PAF‐mediated inflammation and thrombosis in AIS pathogenesis are warranted.

## 5 CONCLUSION

We found that DGMI plus conventional antiplatelet agents were a promising intensive antiplatelet strategy in AIS without any increase in bleeding events compared with patients who received conventional antiplatelet therapy. The analysis integrating transcriptomic and pharmacology identified that DGMI exerts a therapeutic effect in patients with AIS via inflammatory and thrombosis‐related pathways. Since ischemic stroke has multiple and different underlying etiologies, PAFR‐antagonist‐based intensive antiplatelet strategy might shed a new light on the prevention and treatment of AIS.

## AUTHOR CONTRIBUTIONS

Qingyu Shen and Yamei Tang: study concept and design, project administration, and funding acquisition; Xiaoyan Han and Youjia Li: investigation, methodology, funding acquisition, and drafted the manuscript; Xuemin Chen: acquisition, analysis, or interpretation of the data and drafted the manuscript; Dong Pan and Junning Mo: analysis and methodology; Jiaming Qiu: validation; Yi Li: validation and funding acquisition; Yan Chen, and Yan Huang: supervising the study and writing review. All authors critically revised the manuscript. All authors had full access to all the data in the study. All authors have read and approved the submitted version.

## FUNDING INFORMATION

This study was funded by the National Natural Science Foundation of China (8192503181820108026) and the Science and Technology Program of Guangzhou (202007030001) to Yamei Tang; Guangzhou Municipal Science and Technology Project (201904010314) to Qingyu Shen; the Medical Scientific Research Foundation of Guangdong Province of China (B2020154) to Xiaoyan Han; Special Fund for Science and Technology Innovation Strategy in Guangdong Province of China (2018N020) to Youjia Li; National Science and Technology 2030 Program (2022ZD0208900) to Yi Li.

## CONFLICT OF INTEREST STATEMENT

The authors declare there are no potential conflicts of interest with respect to the research, authorship, and/or publication of this article.

## PATIENT CONSENT STATEMENT

The patients' written informed consent was waived because all data analyzed in this study were anonymized.

## Supporting information


Figure S1.

Figure S2.

Figure S3.

Table S1.

Table S2.

Table S3.

## Data Availability

The data used and analyzed during the current study are available from the corresponding author upon reasonable request.
