# Supplementary figures and images for: Platelet‐activating factor antagonist‐based intensive antiplatelet strategy in acute ischemic stroke: A propensity score matched with network pharmacology analysis
Source: CNS Neurosci Ther. 2023 Jul 12;29(12):4082–92. doi: 10.1111/cns.14331 (PMC10651968; doi:10.1111/cns.14331)

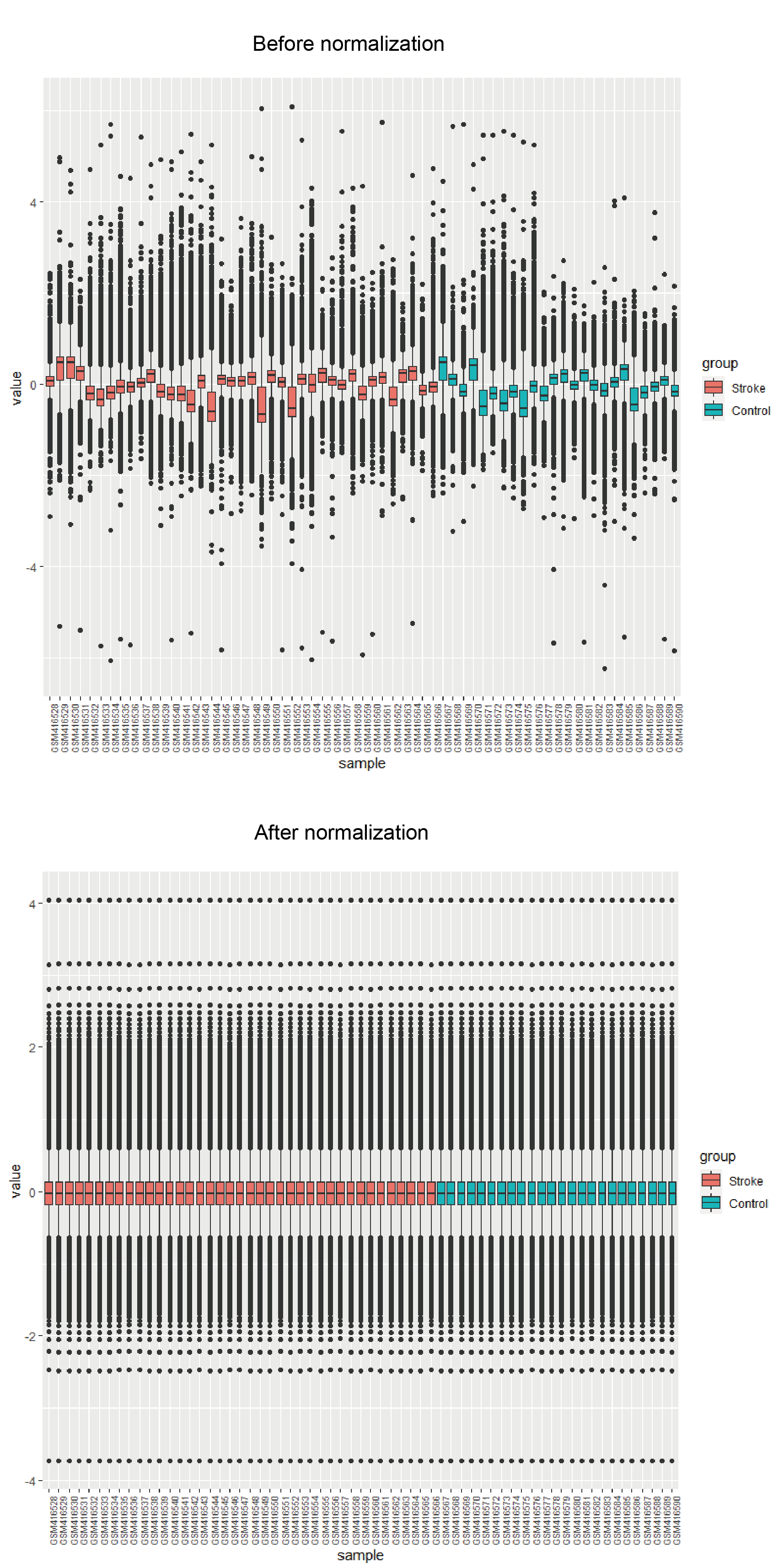

Supplement: Supplementary file 1 — Figure S1. [file CNS-29-4082-s006.tif]

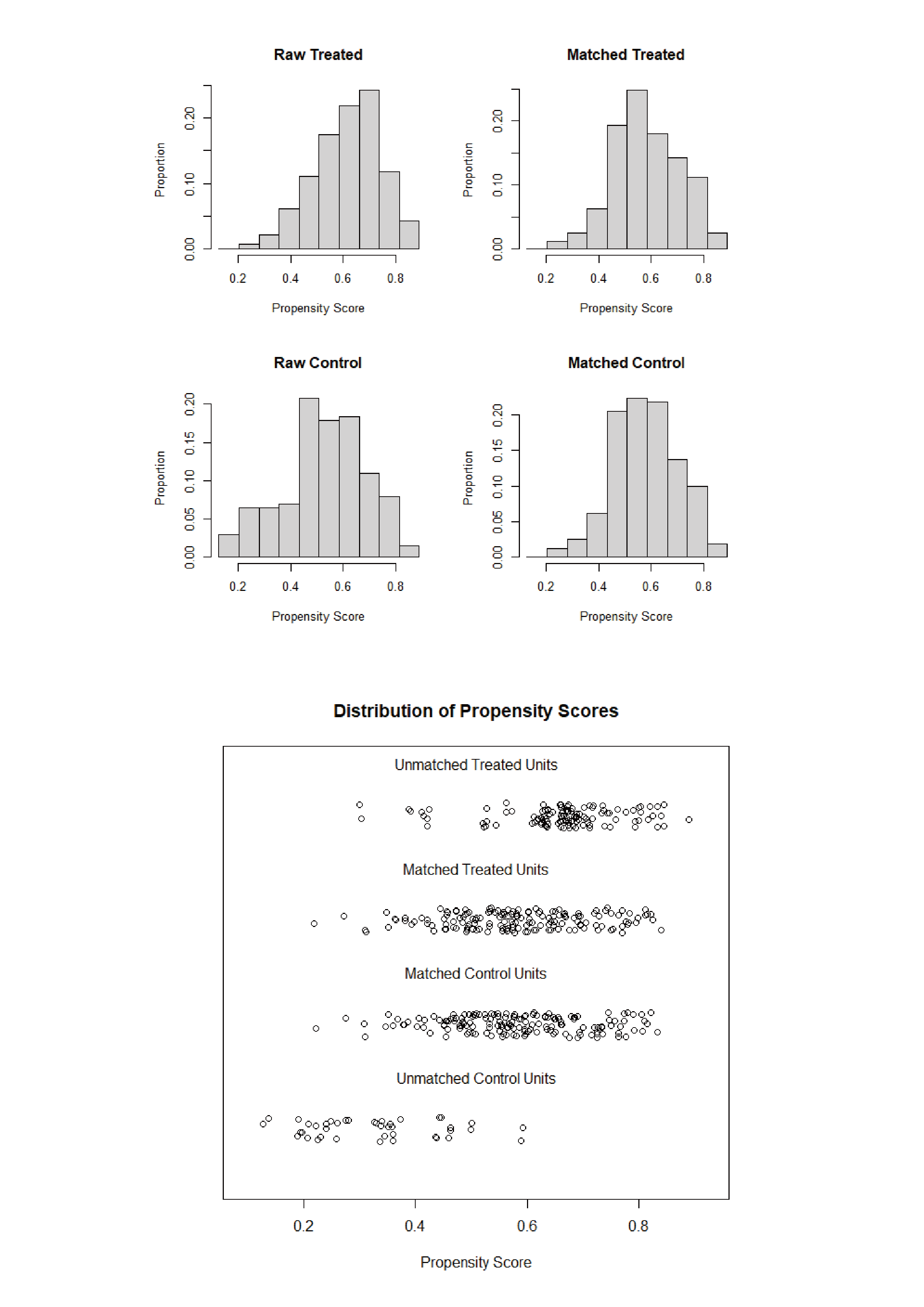

Supplement: Supplementary file 2 — Figure S2. [file CNS-29-4082-s005.tif]

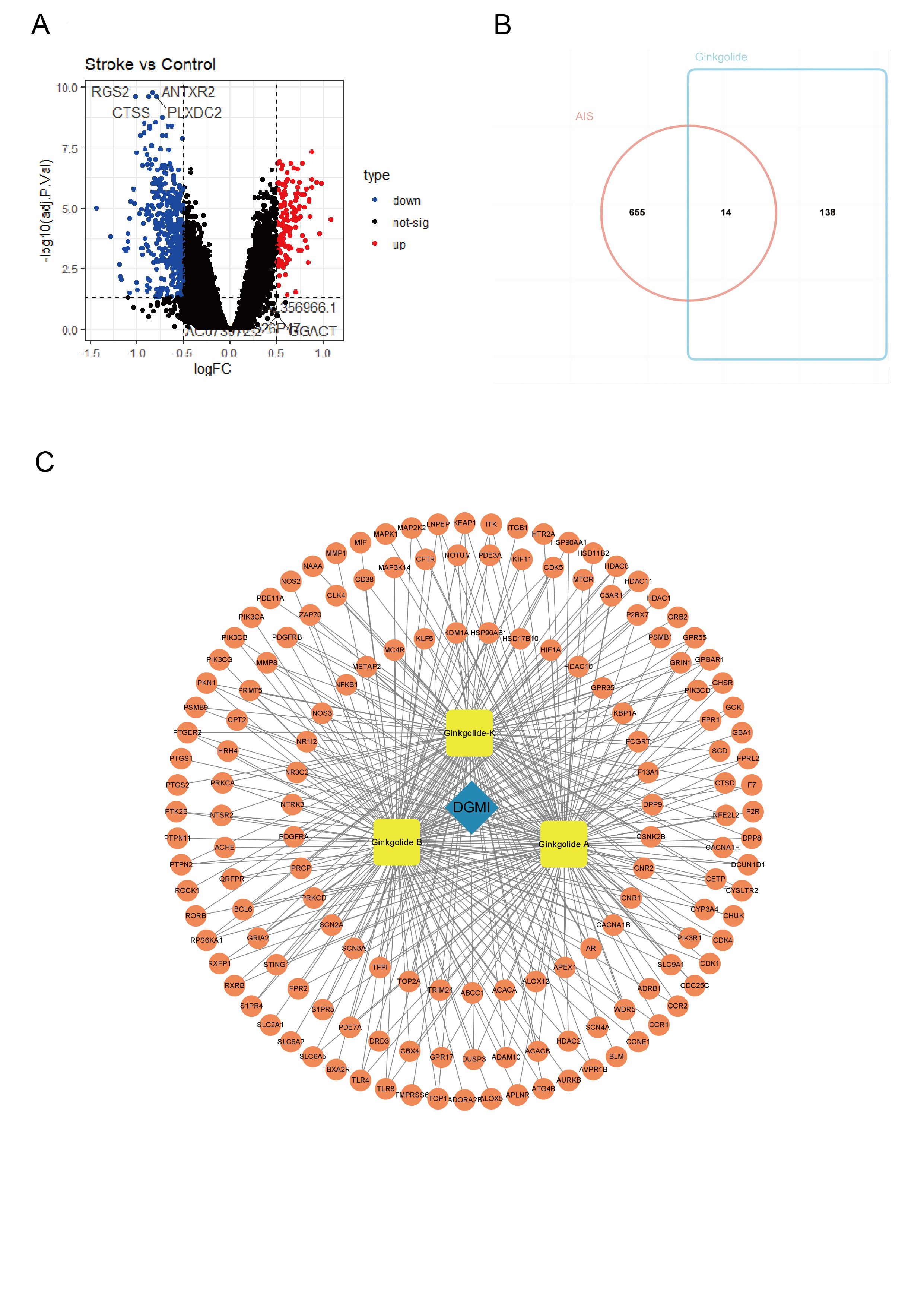

Supplement: Supplementary file 3 — Figure S3. [file CNS-29-4082-s003.tif]
