# Supplementary material for: Platelet‐activating factor antagonist‐based intensive antiplatelet strategy in acute ischemic stroke: A propensity score matched with network pharmacology analysis
Source: CNS Neurosci Ther. 2023 Jul 12;29(12):4082–92. doi: 10.1111/cns.14331 (PMC10651968; doi:10.1111/cns.14331)
Supplement: Supplementary file 4 — Table S1. [file CNS-29-4082-s001.docx]

Supplementary Table 1 Platelet function indicators of patients after treatment.

| Outcomes | non-DGMI  n=161 | DGMI  n=161 | p-value |
| --- | --- | --- | --- |
| R, Median (IQR),min | 6.00 (5.04, 7.00) | 5.80 (5.20, 6.90) | 0.611 |
| K, Median (IQR),min | 2.00 (1.60, 2.38) | 1.90 (1.50, 2.30) | 0.139 |
| angle, Median (IQR), degree | 62.40 (57.80, 66.97) | 62.70 (58.40, 67.90) | 0.193 |
| MA, Mean (SD) | 64.2 ± 7.1 | 64.5 ± 7.1 | 0.503 |
| MAADP, Mean (SD) | 46.4 ± 16.2 | 47 ± 16.4 | 0.457 |
| ADP%, Median (IQR) | 38.70 (19.12, 61.88) | 36.60 (17.90, 61.70) | 0.807 |
| AA%, Median (IQR) | 91.45 (56.40, 100.00) | 91.80 (60.50, 100.00) | 0.683 |

Abbreviations: DGMI, Diterpene ginkgolides meglumine injection; R, reaction time; K, coagulation time; MA, maximum amplitude; MAADP, the adenosine diphosphate induced blood clot strength; ADP%, adenosine diphosphate inhibition rate; AA%, arachidonic acid inhibition rate.
