# Supplementary material for: Platelet‐activating factor antagonist‐based intensive antiplatelet strategy in acute ischemic stroke: A propensity score matched with network pharmacology analysis
Source: CNS Neurosci Ther. 2023 Jul 12;29(12):4082–92. doi: 10.1111/cns.14331 (PMC10651968; doi:10.1111/cns.14331)
Supplement: Supplementary file 5 — Table S2. [file CNS-29-4082-s002.docx]

Supplementary Table 2 The top 20 terms enriched in GO enrichment analysis.

| ONTOLOGY | ID | Description | GeneRatio | BgRatio | p.adjust | qvalue/FDR | geneID |
| --- | --- | --- | --- | --- | --- | --- | --- |
| BP | GO:0002430 | complement receptor mediated signaling pathway | 3/14 | 13/18723 | 0.000105663 | 0.0000438 | C5AR1/FPR2/FPR1 |
| BP | GO:0050727 | regulation of inflammatory response | 6/14 | 386/18723 | 0.000107532 | 0.0000446 | BCL6/FPR2/TLR4/ALOX5/PTGS2/PIK3CG |
| BP | GO:0032103 | positive regulation of response to external stimulus | 6/14 | 427/18723 | 0.000129867 | 0.0000538 | C5AR1/TLR8/FPR2/TLR4/PTGS2/PIK3CG |
| BP | GO:0001819 | positive regulation of cytokine production | 6/14 | 467/18723 | 0.000133434 | 0.0000553 | C5AR1/ITK/TLR8/TLR4/PTGS2/PIK3CG |
| BP | GO:0014002 | astrocyte development | 3/14 | 43/18723 | 0.000603691 | 0.000250196 | C5AR1/FPR2/TLR4 |
| BP | GO:2000377 | regulation of reactive oxygen species metabolic process | 4/14 | 157/18723 | 0.000603691 | 0.000250196 | PRCP/FPR2/TLR4/ALOX5 |
| BP | GO:0050900 | leukocyte migration | 5/14 | 369/18723 | 0.000603691 | 0.000250196 | C5AR1/FPR2/ROCK1/ALOX5/PIK3CG |
| BP | GO:0042060 | wound healing | 5/14 | 422/18723 | 0.000974623 | 0.000403927 | PRCP/TLR4/ALOX5/F13A1/PIK3CG |
| BP | GO:0002274 | myeloid leukocyte activation | 4/14 | 223/18723 | 0.001428063 | 0.000591853 | C5AR1/FPR2/TLR4/PIK3CG |
| BP | GO:0043410 | positive regulation of MAPK cascade | 5/14 | 480/18723 | 0.001428063 | 0.000591853 | C5AR1/FPR2/TLR4/ROCK1/PIK3CG |
| BP | GO:0030595 | leukocyte chemotaxis | 4/14 | 230/18723 | 0.001498642 | 0.000621103 | C5AR1/FPR2/ALOX5/PIK3CG |
| BP | GO:0072593 | reactive oxygen species metabolic process | 4/14 | 239/18723 | 0.001633425 | 0.000676964 | PRCP/FPR2/TLR4/ALOX5 |
| BP | GO:0071260 | cellular response to mechanical stimulus | 3/14 | 81/18723 | 0.001699454 | 0.000704329 | TLR8/TLR4/PTGS2 |
| BP | GO:0072376 | protein activation cascade | 2/14 | 12/18723 | 0.002002409 | 0.000829887 | PRCP/F13A1 |
| BP | GO:0090594 | inflammatory response to wounding | 2/14 | 15/18723 | 0.002747719 | 0.001138776 | TLR4/ALOX5 |
| BP | GO:0007200 | phospholipase C-activating G protein-coupled receptor signaling pathway | 3/14 | 104/18723 | 0.002810053 | 0.00116461 | C5AR1/FPR2/FPR1 |
| BP | GO:0002526 | acute inflammatory response | 3/14 | 112/18723 | 0.002880253 | 0.001193704 | PRCP/PTGS2/PIK3CG |
| BP | GO:0007204 | positive regulation of cytosolic calcium ion concentration | 4/14 | 319/18723 | 0.002880253 | 0.001193704 | C5AR1/FPR2/FPR1/PIK3CG |
| BP | GO:0010631 | epithelial cell migration | 4/14 | 357/18723 | 0.003881058 | 0.001608482 | PRCP/ROCK1/PTGS2/PIK3CG |
| BP | GO:0045089 | positive regulation of innate immune response | 3/14 | 131/18723 | 0.003881058 | 0.001608482 | TLR8/FPR2/TLR4 |
| CC | GO:0101002 | ficolin-1-rich granule | 4/14 | 185/19550 | 0.000292596 | 0.0001567 | PRCP/FPR2/FPR1/ALOX5 |
| CC | GO:0101003 | ficolin-1-rich granule membrane | 3/14 | 61/19550 | 0.000292596 | 0.0001567 | PRCP/FPR2/FPR1 |
| CC | GO:0034774 | secretory granule lumen | 3/14 | 322/19550 | 0.010486796 | 0.005616197 | ROCK1/ALOX5/F13A1 |
| CC | GO:0060205 | cytoplasmic vesicle lumen | 3/14 | 325/19550 | 0.010486796 | 0.005616197 | ROCK1/ALOX5/F13A1 |
| CC | GO:0031983 | vesicle lumen | 3/14 | 327/19550 | 0.010486796 | 0.005616197 | ROCK1/ALOX5/F13A1 |
| CC | GO:0005765 | lysosomal membrane | 3/14 | 395/19550 | 0.014389648 | 0.007706367 | PRCP/TLR8/FPR1 |
| CC | GO:0098852 | lytic vacuole membrane | 3/14 | 395/19550 | 0.014389648 | 0.007706367 | PRCP/TLR8/FPR1 |
| CC | GO:0005766 | primary lysosome | 2/14 | 155/19550 | 0.023408988 | 0.01253667 | PRCP/FPR1 |
| CC | GO:0001726 | ruffle | 2/14 | 178/19550 | 0.027130977 | 0.014529979 | TLR4/ROCK1 |
| CC | GO:0005641 | nuclear envelope lumen | 1/14 | 10/19550 | 0.027130977 | 0.014529979 | ALOX5 |
| MF | GO:0004875 | complement receptor activity | 3/14 | 12/18368 | 0.00000525 | 0.00000236 | C5AR1/FPR2/FPR1 |
| MF | GO:0016702 | oxidoreductase activity, acting on single donors with incorporation of molecular oxygen, incorporation of two atoms of oxygen | 2/14 | 23/18368 | 0.002285083 | 0.001025811 | ALOX5/PTGS2 |
| MF | GO:0016701 | oxidoreductase activity, acting on single donors with incorporation of molecular oxygen | 2/14 | 24/18368 | 0.002285083 | 0.001025811 | ALOX5/PTGS2 |
| MF | GO:0038187 | pattern recognition receptor activity | 2/14 | 26/18368 | 0.002285083 | 0.001025811 | TLR8/TLR4 |
| MF | GO:0003953 | NAD+ nucleosidase activity | 2/14 | 28/18368 | 0.002285083 | 0.001025811 | TLR8/TLR4 |
| MF | GO:0001540 | amyloid-beta binding | 2/14 | 84/18368 | 0.015424489 | 0.006924306 | FPR2/TLR4 |
| MF | GO:0008528 | G protein-coupled peptide receptor activity | 2/14 | 147/18368 | 0.030390868 | 0.013642959 | FPR2/FPR1 |
| MF | GO:0001653 | peptide receptor activity | 2/14 | 153/18368 | 0.030390868 | 0.013642959 | FPR2/FPR1 |
| MF | GO:0008239 | dipeptidyl-peptidase activity | 1/14 | 10/18368 | 0.030390868 | 0.013642959 | PRCP |
| MF | GO:0016303 | 1-phosphatidylinositol-3-kinase activity | 1/14 | 10/18368 | 0.030390868 | 0.013642959 | PIK3CG |
