# Supplementary material for: Platelet‐activating factor antagonist‐based intensive antiplatelet strategy in acute ischemic stroke: A propensity score matched with network pharmacology analysis
Source: CNS Neurosci Ther. 2023 Jul 12;29(12):4082–92. doi: 10.1111/cns.14331 (PMC10651968; doi:10.1111/cns.14331)
Supplement: Supplementary file 6 — Table S3. [file CNS-29-4082-s004.docx]

Supplementary Table 3 The terms enriched in Reactome enrichment analysis.

| ID | Description | GeneRatio | BgRatio | P.adjust | qvalue/FDR | geneID |
| --- | --- | --- | --- | --- | --- | --- |
| R-HSA-6785807 | Interleukin-4 and Interleukin-13 signaling | 4/14 | 108/10899 | 0.000828085 | 0.000414677 | BCL6/ALOX5/F13A1/PTGS2 |
| R-HSA-6798695 | Neutrophil degranulation | 6/14 | 482/10899 | 0.000828085 | 0.000414677 | C5AR1/PRCP/FPR2/FPR1/ROCK1/ALOX5 |
| R-HSA-9018677 | Biosynthesis of DHA-derived SPMs | 2/14 | 17/10899 | 0.005330649 | 0.00266941 | ALOX5/PTGS2 |
| R-HSA-449147 | Signaling by Interleukins | 5/14 | 473/10899 | 0.005330649 | 0.00266941 | BCL6/FPR1/ALOX5/F13A1/PTGS2 |
| R-HSA-9018678 | Biosynthesis of specialized proresolving mediators (SPMs) | 2/14 | 19/10899 | 0.005330649 | 0.00266941 | ALOX5/PTGS2 |
| R-HSA-140877 | Formation of Fibrin Clot (Clotting Cascade) | 2/14 | 39/10899 | 0.018969061 | 0.00949907 | PRCP/F13A1 |
| R-HSA-6783783 | Interleukin-10 signaling | 2/14 | 47/10899 | 0.023580736 | 0.011808442 | FPR1/PTGS2 |
| R-HSA-375276 | Peptide ligand-binding receptors | 3/14 | 201/10899 | 0.024923801 | 0.012481004 | C5AR1/FPR2/FPR1 |
| R-HSA-2142753 | Arachidonic acid metabolism | 2/14 | 59/10899 | 0.028774936 | 0.014409524 | ALOX5/PTGS2 |
